# A genetic model for central chondrosarcoma evolution correlates with patient outcome

**DOI:** 10.1186/s13073-022-01084-0

**Published:** 2022-08-30

**Authors:** William Cross, Iben Lyskjær, Tom Lesluyes, Steven Hargreaves, Anna-Christina Strobl, Christopher Davies, Sara Waise, Shadi Hames-Fathi, Dahmane Oukrif, Hongtao Ye, Fernanda Amary, Roberto Tirabosco, Craig Gerrand, Toby Baker, David Barnes, Christopher Steele, Ludmil Alexandrov, Gareth Bond, Genomics England Research Consortium, Paul Cool, Nischalan Pillay, Peter Van Loo, Adrienne M. Flanagan

**Affiliations:** 1 Research Department of Pathology, University College London, UCL Cancer Institute, London, UK; 2 Medical Genomics Research Group, University College London, UCL Cancer Institute, London, UK; 3 The Francis Crick Institute, London, UK; 4 Department of Histopathology, Royal National Orthopaedic Hospital, Stanmore, UK; 5 Cancer Sciences Unit, University of Southampton, Southampton, UK; 6 Bone Tumour Unit, Royal National Orthopaedic Hospital, Stanmore, UK; 7 Institute of Cancer and Genomic Sciences, Birmingham University, Birmingham, UK; 8 University of California, San Diego, USA; 9 Genomics England Limited, London, UK; 10 Robert Jones & Agnes Hunt Orthopaedic Hospital NHS Foundation Trust, Oswestry, UK; 11 Keele University, Keele, UK

**Keywords:** Chondrosarcoma, Sarcoma, Genetics, Genomics, Cancer evolution, IDH1, IDH2, TERT

## Abstract

**Background:**

Central conventional chondrosarcoma (CS) is the most common subtype of primary malignant bone tumour in adults. Treatment options are usually limited to surgery, and prognosis is challenging. These tumours are characterised by the presence and absence of *IDH1* and *IDH2* mutations, and recently, *TERT* promoter alterations have been reported in around 20% of cases. The effect of these mutations on clinical outcome remains unclear. The purpose of this study was to determine if prognostic accuracy can be improved by the addition of genomic data, and specifically by examination of *IDH1, IDH2*, and *TERT* mutations.

**Methods:**

In this study, we combined both archival samples and data sourced from the Genomics England 100,000 Genomes Project (*n* = 356). Mutations in *IDH1*, *IDH2*, and *TERT* were profiled using digital droplet PCR (*n* = 346), whole genome sequencing (*n*=68), or both (*n* = 64). Complex events and other genetic features were also examined, along with methylation array data (*n* = 84). We correlated clinical features and patient outcomes with our genetic findings.

**Results:**

*IDH2*-mutant tumours occur in older patients and commonly present with high-grade or dedifferentiated disease. Notably, *TERT* mutations occur most frequently in *IDH2*-mutant tumours, although have no effect on survival in this group. In contrast, *TERT* mutations are rarer in *IDH1*-mutant tumours, yet they are associated with a less favourable outcome in this group. We also found that methylation profiles distinguish *IDH1-* from *IDH2*-mutant tumours. IDH wild-type tumours rarely exhibit *TERT* mutations and tend to be diagnosed in a younger population than those with tumours harbouring *IDH1* and *IDH2* mutations. A major genetic feature of this group is haploidisation and subsequent genome doubling. These tumours evolve less frequently to dedifferentiated disease and therefore constitute a lower risk group.

**Conclusions:**

Tumours with *IDH1* or *IDH2* mutations or those that are *IDHwt* have significantly different genetic pathways and outcomes in relation to *TERT* mutation. Diagnostic testing for *IDH1*, *IDH2*, and *TERT* mutations could therefore help to guide clinical monitoring and prognostication.

**Supplementary Information:**

The online version contains supplementary material available at 10.1186/s13073-022-01084-0.

## Background

Central conventional chondrosarcoma (CS) is the most common primary malignant bone tumour in adults. Anatomical location, histopathology, and grading are the current criteria for determining treatment [1, 2], although providing prognoses remains challenging [1–3]; hence, a greater understanding of the disease, and its biomarkers is required to provide patients with a more personalised treatment plan. Well-differentiated cartilaginous tumours are referred to differently depending on where they develop: those presenting at sites, from where they can be excised fully with relative ease, including the phalanges and long bones they have an excellent prognosis, and are referred to as atypical cartilaginous tumours (ACT), the exception being if they are associated with a dedifferentiated (DD) component. In contrast, tumours with features of ACT that occur at sites where complete excision is difficult, such as the axial skeleton and pelvis, are referred to as CS grade (G) 1; these lesions often recur locally and are associated with transformation to higher tumour grade, with many patients eventually succumbing to their disease [4, 5]. G2 disease represents approximately 40% of central CS, and has a 5-year survival of approximately 70–99%, whereas G3 disease, comprising about 10% of all central CS, has a 5-year survival of approximately 30–77% [4, 5]. The most aggressive form of the disease is the DD subtype, which arises on the background of about 10% of all conventional central chondrosarcomas and has a 5-year survival of 7–24% [2, 4, 5]. In contrast high grade disease of the phalanges is uncommon and has little metastatic potential; DD CS rarely occurs at this site [6].

The cytosolic isocitrate dehydrogenase type 1 (*IDH1*) and mitochondrial isocitrate dehydrogenase type 2 (*IDH2*) enzymes are key components in the tricarboxylic acid cycle. Specific alterations at the R132 and R172 amino acid residues of these genes respectively occur in CS (amongst other cancers), disrupting normal functions and leading to the accumulation of 2-D-hydroxyglutarate (2HG), a competitive inhibitor of alpha KG-dependent dioxygenases. This results in downstream affects, including hypermethylation of CpG islands [7].

Close to 70% of central CS harbour an *IDH1* (60%) or an *IDH2* (10%) mutation [8], and these are considered key initiators of disease [8–11]. No recurrent initiating genetic drivers have been reported in the remaining 30% of *IDH1/2* wild type (IDHwt) cases [12, 13], although these tumours have been reported to exhibit different methylation profiles compared to *IDH1* and *IDH2*-mutant tumours [14, 15], hereafter referred to as *IDH1* and *IDH2* tumours. Other key drivers include mutations in *COL2A1*, *CDKN2A/B*, and *TP53* [12, 13, 16, 17] as well as less common pathogenic alterations in cell cycle-related genes such as *RB1* [18] and *CDK4*/*6* [12, 19], alterations in the Indian Hedgehog pathway, and amplifications of *MYC* [12, 13]. Alterations in *CDKN2A* and *TP53* are more likely to occur in high grade disease (G2, G3, and DD CS) [12, 17, 19]. These alterations have limited value as markers for survival or risk stratification. Previously, near haploid karyotypes have been reported in CS [20–23], although the relationship with other mutations has not been reported.

The recently identified alterations in the *TERT* promoter locus (C228T) are thought to result in increased telomerase expression leading to immortalisation. These mutations rarely occur in well differentiated tumours, meaning they are a reliable prognostic marker for CS [24]. In contrast, the impact of *IDH1* and *IDH2* mutations on survival remains unclear [9, 25, 26] possibly reflecting the relatively small number of cases studied.

The aim of this study was to undertake a comprehensive analysis of a large set of CS genomes, leveraging data from the Genomics England 100,000 Genomes Project [27] combined with digital droplet PCR (ddPCR) and methylation profiling. A particular focus of our efforts was to identify mutations of relevance to patient outcome and identify recurrent driver mutations in those tumours that are wild type for *IDH1* and *IDH2* mutations (IDHwt).

## Methods

### Patients and samples

Three hundred fifty-six cases of CS were included in the study. No enchondromas were included. These included 68 tumour-normal paired samples from four clinical sites (Royal National Orthopaedic Hospital Stanmore, Royal Orthopaedic Hospital Birmingham, Nottingham NHS Trust, Queen Elizabeth Hospital Birmingham) that were subjected to whole genome sequencing (WGS) as part of the Genomics England 100,000 Genomes Project (hereby referred to as the *100KGP* cohort, Additional file 1: Supplementary Table 1-2). The remaining cases were obtained from the archives of the Royal National Orthopaedic Hospital, Stanmore (Additional file 1: Supplementary Table 3). The pathology was reviewed Adrienne Flanagan, Fernanda Amary, and Roberto Tirabosco. For cases with clinical follow-up, the median surveillance time was 5.6 years (2059 days, range: 5–10,057 days). Nearly all patients received surgery as standard of care; two died prior to receiving definitive treatment, and 6% (*n* = 22) of patients received adjuvant treatment in the form of doxorubicin/cisplatin or radiotherapy.

### Bioinformatic pre-processing and statistical assessment

Single nucleotide variants (SNVs) and indels were called on WGS data and filtered using a panel of normal (PON) samples via the Genomics England analysis pipeline, which utilises Strelka and other tools [28] (Additional file 2: Supplementary Methods). To quality assess the 100KGP mutation calls, we performed orthogonal verification of hotspot mutations in *IDH1* (R132), *IDH2* (R172), and *TERT* (C228T) identified across 64 patients (59 mutations in total) using ddPCR, which yielded a recall rate of 100%. There was one instance (WGS_53) where a mutation was called by ddPCR but not in the WGS data (*IDH1* ddPCR, IDHwt WGS, later result used, Additional file 2: Supplementary Methods). Somatic copy number variants were called using Battenberg [29] and structural variants (SVs) were called using Delly (v0.8.5) [30]. Unless otherwise specified, comparisons between groups were performed using Wilcoxon tests for distributions and Fisher exact tests for group counts (i.e. in *IDH1*/*2*/WT group comparisons). Survival analysis utilised a Kaplan-Meier standard Cox proportional hazard model.

### Identification of driver mutations, genome doubling, partial haploidisation, and analysis of mutational signatures

Driver mutations in SNVs and indels were identified using a combination of known hotspot locations published previously and available in Additional file 2: Supplementary Methods, the SIFT [31] and POLYPHEN [32] tools, plus visual inspection using integrative genomics viewer (IGV) of the *IDH1* R132, *IDH2* R172, and *TERT* mutations (Additional file 3: Supplementary Fig. 1, Note 1). Amplification events were designated as copy states of five or in diploid genomes and nine or more in those that are genome doubled. For the purposes of plotting, we classified copy states from Battenberg as either, diploid, trisomy or tetrasomy, copy neutral LOH (cnLOH), and the remaining copy states as ‘other’ (any remaining copy state). Tumours with genome doubling (GD) were identified using a clustering procedure based on the R package Mclust [33]. Cases with more than 50% LOH were marked as exhibiting partial haploidisation. We confirmed the ploidy status in 14 of the 100KGP cases using flow cytometry (Additional file 2: Supplementary Methods, Additional file 3: Supplementary Fig. 2, Note 2). To time the appearance of GD, we used a methodology based on molecular-clock principles [34, 35]. Ninety-six channel single-base substitution (SBS) mutational signatures were extracted from the Strelka-called SNVs using SigProfilerExtractor [36] version 1.1.3 with default parameters.

### Methylation data protocol and analysis

We analysed 84 cases using methylation arrays (Additional file 3: Supplementary Tables 4-6, Supplementary Fig. 3). Five hundred nanograms of DNA from frozen tumour samples were bisulphite converted using Zymo EZ DNA methylation Gold kit (Zymo Research Corporation Irvine, CA, USA) and hybridised to the Infinium HumanMethylationEPIC beadchip arrays (Illumina, San Diego, CA). The generated methylation data were analysed using the ChAMP R [37], normalised using BMIQ, and hierarchical clustering plots were constructed using the ‘pheatmap’ R package [38].

*TERT* promoter methylation status was determined by the methylation status of the cg11625005 probe as reported previously [39]. Raw DNA methylation data files have been deposited in the ArrayExpress database (https://www.ebi.ac.uk/arrayexpress/experiments/E-MTAB-11031/, accession: E-MTAB-11031).

### Statistics and mathematical analysis

In all group comparison situations, such as *IDH1*, *IDH2*, and IDH-WT cases, with or without *TERT* mutations, we used Fisher test statistics as implemented in R (testing both 3X2 and 2x2 contingency tables). Distributions of data, as seen in the tests of timing for GD was performed using Wilcoxon tests, again implemented in R. Linear regressions were also implemented using the standard R methods. The cox proportional hazard model and Kaplan-Meyer, were obtained via the *survminer* package [40]. For the chromosome arm frequency comparisons, we used Fisher tests and the Bonferroni multiple testing correction. For power calculations, please see Additional file 2: Supplementary Methods.

## Results

### Driver mutations in central conventional and dedifferentiated chondrosarcomas

Profiling a total of 350 CS cases for *IDH1* and *IDH2*, using ddPCR (*n* = 282) and WGS (*n* = 68), we verified previous findings that *IDH2* are less frequent than *IDH1* mutations (*IDH1*: 51%, *IDH2*: 14%, IDHwt: 35%) [16, 26, 41]. In cases with grading information (*n* = 343), we found that *IDH1* mutations were equally frequent across grades (ACT/G1: 51%, G2/3: 53%, DD CS: 55%; *p* = 0.9) and that *IDH2* mutations were more frequent in higher grade disease (ACT/G1: 7%, G2/3: 14%, DD: 25%; *p* = 0.005). IDHwt was negatively associated with increasing grade (%IDHwt; ACT/G1: 41%, G2/3: 32%, DD CS: 19%; *p* = 0.01). These data imply that the progression to DD CS is more common in tumours with *IDH2* mutations and least common in IDHwt tumours.

**Fig. 1:**
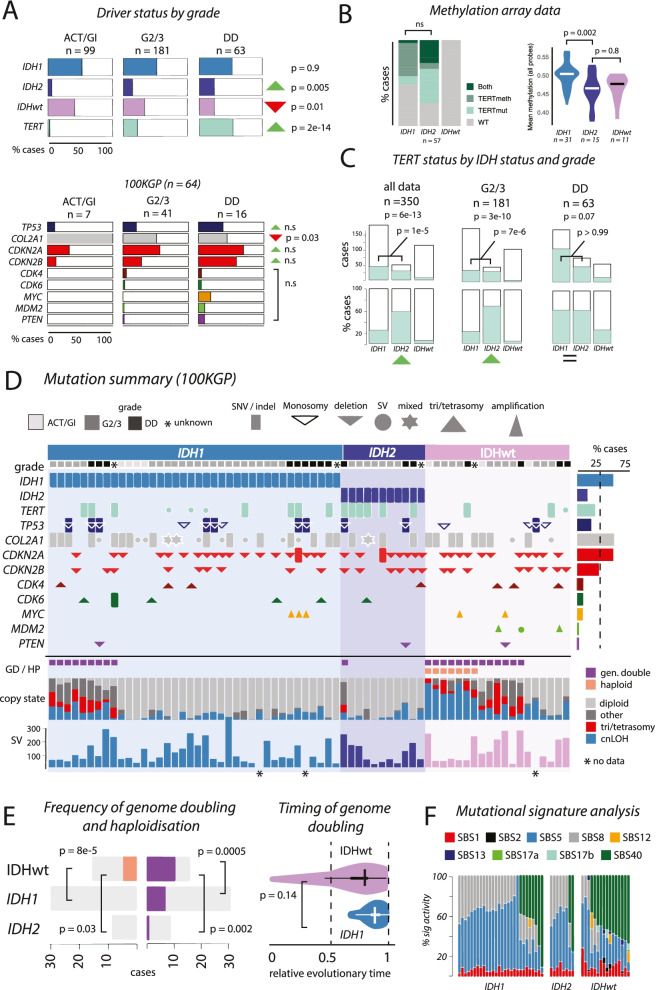
Summary of Genomics England Cohort. **A** Summary of driver mutations by grade. *IDH1* mutations are frequent across all grades, although *IDH2* and *TERT* mutations are enriched in G2/3 and DD CS tumours. **B** *TERT* mutation and methylation status (left) and overall genomic methylation levels (right) across IDH groups. **C** *TERT* mutation status across IDH groups. Canonical *TERT* promoter mutations are common in *IDH2***-**mutant tumours but rare in IDHwt tumours (left plot). In G2/3 tumours, *TERT* alterations are more common in *IDH2* compared to *IDH1* tumours (middle plot), though equally common in DD CS (right plot). *p*-values for tests across all IDH groups above, *IDH1* vs *IDH2* are marked on plots. **D** Mutational calling showing driver calls, genome doubling (GD) and haploidisation (HP), Battenberg copy states (diploid, gain, copy neutral LOH, and any other copy state), and Delly structural variant calls (methods). **E** GD and HP overview by IDH status. Timing of GD shown on right (Additional file 1: Supplementary Methods). **F** Mutational signature analysis demonstrating commonality of SBS2, 5, and 8, and prominence of SBS40 in IDH groups.

Canonical mutations and structural changes near the *TERT* promoter have been reported in approximately 20% of CS and found to correlate with high grade disease [24, 26, 42]. We found a similar frequency in our cohort (23%, 74 with C228T, one with C250T, four with structural changes near the *TERT* promoter (Additional file 3: Supplementary Fig. 1). *TERT* mutations were rare in well differentiated tumours and increased in frequency across grades (ACT/G1: 3%, G2/3: 22%, DD: 56%; *p* = 2e-14, Fig. 1A). We found that the *TERT* promoter was hypermethylated in 19/57 (33%) of cases analysed on methylation arrays, excluding DD CS cases (Fig. 1B). Seven of these cases, all high grade, also harboured *TERT* C228T promoter mutations. There was no significant difference between the number of cases with both *TERT* promoter mutations and hypermethylation across *IDH1* and *IDH2* tumours (Fig. 1B). Rare alterations involving *ATRX* have been reported previously [26], but in the 100KGP cohort (*n* = 68), we found no such alterations in this cohort. These data confirm that *TERT* mutations, and possibly methylation, have distinct roles in CS progression.

We next examined mutations in other key driver genes reported in CS, utilising the 100KGP cohort (Fig. 1A)*.* Our findings were largely similar to those previously reported [13, 43]. Monosomies of 17p and pathogenic SNVs/indels in *TP53* were found in 22% of cases in line with previous reports [12]. *TP53* mutations were absent from all but one well-differentiated tumour. *COL2A1* mutations were common and marginally anti-correlated with increasing grade (ACT/G1: 100%, G2/3: 54%, DD CS: 44%; *p* = 0.03). As previously reported [17, 19, 21], pathogenic SNVs/indels and/or bi-allelic deletions of *CDKN2A* and *CDKN2B* were common in CS and enriched in G2/3 and DD CS, though not significantly in this dataset. Hypermethylation of these genes was not detected. *CDK4* and *CDK6* gains were found in 12 cases, and a single case had a pathogenic SNV in *CDK6*. These frequencies are similar to previous reports [12]. *MYC* amplifications were found in five high-grade tumours establishing its status as a driver of CS [44, 45]. *MDM2* alterations were identified in three high grade IDHwt cases, two of which were amplifications (one 8 copies, one 31 copies, confirmed using fluorescence in situ hybridisation), and one was a structural alteration involving intron 7 of *MDM2* and an intragenic region on chr4q28.3. The latter did not result in amplification of *MDM2* but removal of the zinc finger binding domains, which has been suggested to have an oncogenic effect [46]. All three mutations were mutually exclusive of *TP53* mutations. These data support the premise that *MDM2* is a potential driver gene in CS [26], though any biological effects require further exploration. Homozygous deletions of *PTEN* were present in three high grade cases. *PTEN* promoter hypermethylation was found in 13/57 cases, all high grade. Analysis utilising dNdS [47] returned no previously unknown drivers, implying that all prominent somatically mutated genes driving CS have likely been identified (Additional file 2: Supplementary Methods, Additional file 3: Supplementary Fig. 2).

### IDH1, IDH2, and TERT define key genetic subgroups

Analysis of all mutation calls (*n* = 350) revealed that the frequency of *TERT* mutations was different across *IDH1*, *IDH2*, and IDHwt cases (Fig. 1C). *IDH2* mutations were strongly associated with *TERT* mutations (IDHwt: 5%, *IDH1*: 24%, *IDH2*: 58%, *p* = 6e−13; *IDH1 vs IDH2: p* = 1e−5). This association was observed in G2/3 (*IDH1* vs *IDH2: p* = 7e−6) but not in DD CS (*IDH1* vs *IDH2*: *p* > 0.99), implying that although *TERT* is associated with high-grade tumours, this is not equal in the context of IDH mutation status.

### Hypermethylation across IDH1- and IDH2-mutated tumours

CpG island DNA hypermethylation has been reported to distinguish between cartilaginous *IDH* and IDHwt tumours [14, 15]. However, utilising the larger numbers available in this study, we found 3468 differentially methylated probes (DMPs) across *IDH1* and *IDH2* tumours, excluding DD CS (*n* = 31, *p* = 0.002, Additional file 3: Supplementary Fig. 2, Supplementary Tables 4-6). The overall methylation level across all probes also revealed significant differences between *IDH1* and *IDH2* tumours (*p* = 0.002) indicating that the former are globally hypermethylated compared to *IDH2* and IDHwt tumours.

### Partial haploidisation followed by genome doubling is common in IDHwt tumours

We compared the mutational profiles across each IDH group (complete summary of 100KGP data shown in Fig. 1D) and found that the frequency of common drivers, excluding *TERT*, was similar across *IDH1* and *IDH2* and IDHwt tumours. We did not find that mutations in *CDKN2A/B and TP53* were enriched in *IDH1/2* cases, as previously reported [48] but contrasting another study [26]. The total number of SVs was not statistically different across the *IDH* groups nor was the number of SVs that fell into gene regions. We did not find any common structural variants affecting the same gene more than 25% of cases, although none of these were cancer-related genes (Additional file 2: Supplementary Methods).

The genetic alterations initiating development of IDHwt CS remains unknown, but previous reports of near-haploid (HP) and hyperhaploidy in CS and in other sarcoma subtypes including undifferentiated sarcomas and malignant peripheral nerve sheath tumours prompted us to investigate this [21, 22, 49, 50]. We found 23 tumours with GD and seven with HP in the 100KGP cohort (*n*=68, Additional file 3: Supplementary Fig. 4, Additional file 2: Supplementary Methods). Most GD events (16/23, 69%) occurred in the absence of HP, whereas HP always occurred with GD (Fig. 1D). GD was highly enriched in IDHwt tumours (GD%, *IDH1*: 24%, *IDH2*: 9%, IDHwt: 63%, IDHwt vs *IDH1 p* = 0.0005, Fig. 1E), and HP was exclusive to this group (HP%, *IDH1*: 0%, *IDH2*: 0%, IDHwt: 37%, IDHwt vs *IDH1 p* = 8e-5, Fig. 1E). Timing analysis demonstrated that GD events tended to occur at a similar relative time in IDHwt and *IDH1* cases implying that it could be an intermediate or late event in evolutionary timelines of both tumour groups (Fig. 1E, Additional file 2: Supplementary Methods). The six cases of IDHwt tumours without HP/GD events, harboured mutations in *TP53* and *CDKN2A,* although alterations in these genes were not mutually exclusive with the absence of GD and HP (*TP53*: 3/6, 50%, *CDKN2A/B*: 5/6, 83%, Fig. 1D). One of these cases was ACT/G1, pointing to a possible initiating role of *TP53* and *CDKN2A* in some IDHwt tumours.

### Mutational signatures across IDH1, IDH2, and IDHwt groups

Analysis of mutational signatures in the 100KGP cohort (*n* = 52, Fig. 1F, Additional file 3: Supplementary Fig. 5) revealed nine active signals, with SBS1, SBS5, and SBS8 being ubiquitous and most prominent across *IDH1*, *IDH2*, and IDHwt tumours. Five signatures (SBS2, SBS12, SBS13, and SBS17a/b) were principally exclusive to IDHwt tumours. SBS2 and SBS13 have been associated with APOBEC and were simultaneously active in five IDHwt cases (18%). We did not observe any difference in SNV burden in tumours with active SBS2 and SBS13. SBS12 was found in one *IDH1* case and three IDHwt cases. SBS17a/b, signatures with unknown aetiology, were found only in IDHwt cases. SBS40, also of unknown aetiology, was found in 28% of *IDH1* cases, 25% of *IDH2* cases, and 81% of IDHwt. These data demonstrate that *IDH1* and *IDH2* tumours are comparable in terms of mutational signatures, whereas IDHwt tumours exhibit more heterogeneous mutational processes.

### The genetic distinction between central conventional and dedifferentiated chondrosarcoma

We next analysed the DD CS for specific alterations that may explain their histological phenotype and their poor clinical outcomes. We confirmed that metastatic disease was most common in DD CS (60%, compared to 27% in G2/3 and <1% in ACT/G1 (G2/3 vs DD: *p* = 1e−5). Analysing the 100KGP data (DD: *n* = 16, G2/3: *n* = 41), the frequency of identified known drivers in DD CS and G2/3 revealed no difference except for *IDH2* and *TERT*, which were enriched in DD (*IDH2*: *p* = 0.05, *TERT*: *p* = 3e−6). However, we found differences in total driver burden (*p* = 2e−8), SNV burden (*p* = 0.009), number of chromosome segments (*p* = 0.01), and SV burden (*p* = 0.01) (Additional file 3: Supplementary Fig. 6). We next explored whether the increased segment counts were attributable to chromothripsis. Using a previously published method [51], we found only one instance of chromothripsis (WGS_21) which overlapped with the SV identified at the *TERT* loci (Fig. 1D, Additional file 3: Supplementary Fig. 1). Examining the number more broadly, the average number of chromosomes with high breakage was higher in DD CS compared to G2/3 (median, G2/3: 0, DD CS: 2.5, *p* = 0.03, see Additional file 2: Supplementary Methods). There were no specific chromosome arms enriched amongst those with high fragmentation, although three cases (19%) had fragmentation across chromosome 12q, which has also been reported in dedifferentiated liposarcoma [52]. Previous studies have reported that aberrations of chromosome 5q and trisomy of chromosome 19 distinguish G2/3 from DD CS [53]. Twenty-five percent DD CS harboured 19p/q gains which is less than the 50% previously reported [53]. Examining losses and gains across all chromosome arms revealed no events unique to DD CS although losses at 15q were more common in this subtype (15q loss, G2/3, 10%, DD CS: 38%, *p* = 0.05). Together, these analyses suggest that the primary genetic difference between G2/3 and DD CS is the number of accrued SNVs and the degree of chromosomal instability.

### Age at diagnosis as a clinical factor in chondrosarcoma

Previous studies of CS have treated *IDH1* and *IDH2* tumours as one group [14, 54]. Our results, leveraging hundreds of cases, provide evidence that *IDH1* and *IDH2* mutations lead to distinct downstream genetic events, with differences in the frequency of *TERT* mutations, GD/HP, methylation profiles, and the number and types of mutational signatures.

**Fig. 2:**
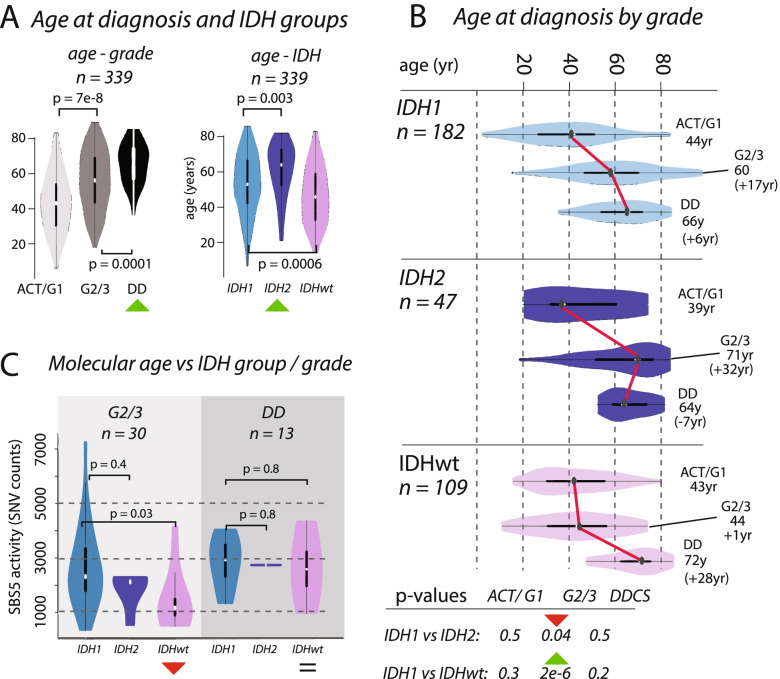
Divergences in chronological and molecular age in chondrosarcoma. **A** Boxplots showing the distribution of age at diagnosis (*n* = 339) increasing across grades. Distributions differ across *IDH1*, *IDH2*, and IDHwt groups, with *IDH2* tumours occurring in older patients compared to those with *IDH1* and IDHwt tumours. **B** Boxplots of age, broken down by IDH status and grade. **C** The differences in chronological age between G2/3 *IDH1* and *IDH2* tumours and IDHwt tumours (**B**) is recapitulated in the activities of mutational signature SBS5. There is no significant difference in molecular age of *IDH1* and *IDH2* tumours, whereas there is a significant difference in the chronological age

We examined the effect of the presence or absence of *IDH1* and *IDH2* mutations on the clinical behaviour of CS (*n* = 339, Fig. 2). We showed that patient age at diagnosis increased across grades in these groups and that the median age was highest in those with *IDH2* tumours (*IDH1*: 55 year, *IDH2*: 67 year, IDHwt: 47 year, *IDH1* vs *IDH2*: *p* = 0.003, *IDH1* vs IDHwt: *p* = 0.0006, Fig. 2A). The age at diagnosis for each IDH group was similar for ACT/G1 and DD CS, and the difference in age of the G2/3 tumours explained the overall difference in ages (median age G2/3, *IDH1*: 60 year, *IDH2*: 71 year, IDHwt: 44 year, *IDH1*vs *IDH2*: *p* = 0.04, *IDH1* vs IDHwt: *p* = 2e−6, Fig. 2B). We considered whether these differences in chronological age at diagnosis were reflected in the mutational signatures active in each group. The total SNV burden correlated with age at diagnosis, as did SBS5, previously been reported as clock-like [55], SBS8, but not SBS1. In G2/3 tumours, the activity of SBS5 was similar in *IDH1* and *IDH2*, but lower in IDHwt (*IDH1* vs *IDH2 p* = 0.4, *IDH1* vs IDHwt: *p* = 0.03, Fig. 2C). By contrast, SBS5 activity was similar in all DD cases (*IDH1* vs *IDH2 p* = 0.7, *IDH1* vs IDHwt: *p* = 0.8, Fig. 2C). These same results were recapitulated when using SBS8 and total SNV burden (Additional file 3: Supplementary Fig. 7). Together, these data imply further differences in the rate of evolution from G2/3 to DD CS across *IDH1*, *IDH2*, and IDHwt tumours.

### Divergent outcomes in IDH1, IDH2 and IDHwt tumours

**Fig. 3:**
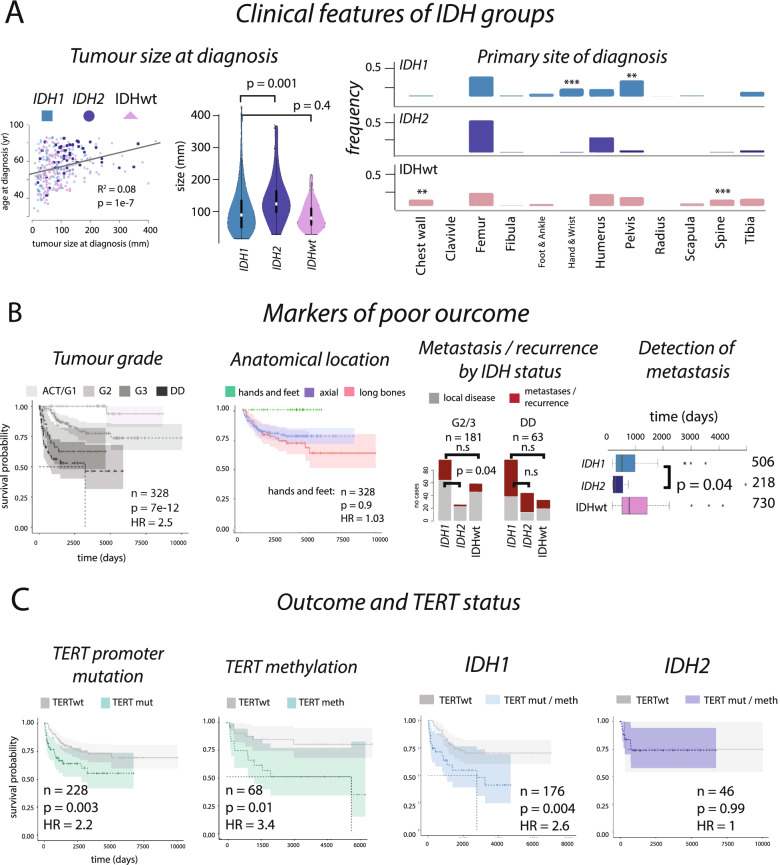
Outcomes in chondrosarcoma. **A** Tumour size of *IDH1*, *IDH2*, and IDHwt CS are different at presentation and their anatomical location are largely comparable, although IDHwt tumours develop more frequently in the chest wall and spine (left and middle, and right, respectively). **B** Kaplan-Meier analysis and hazard ratios (HR) from Cox proportional hazard analysis confirms tumour grade, and anatomical location, as predictors of outcome. The frequency of metastatic/recurrent disease is significantly lower in *IDH2* G2/3 disease compared to *IDH1* G2/3 disease but is comparable in DD CS. The time interval between diagnosis and discovery of metastatic disease is shortest in *IDH2*-driven tumours and on average longest in IDHwt (while also being less frequent). Median time in days given right of plot. **C** *TERT* mutations are linked to poor outcome, as is methylation of *TERT* (left plots). In high-grade (G2/3 and DD CS) *IDH1* tumours, *TERT* mutations associate with a poor outcome, but not in *IDH2* tumours

Using all available clinical information (*n* = 342), we found that *IDH2* tumours tended to be larger at time of presentation (*IDH1* vs *IDH2*: *p* = 0.001, *IDH1* vs IDHwt: *p* = 0.4, Fig. 3A), supporting the premise that these tumours evolve over longer time periods, and present in older people. Development in specific anatomical locations was not significantly different (Fig. 3A).

Using all cases with available follow-up data (*n* = 328), a Cox proportional hazard model demonstrated that ACT/G1 tumours nearly always had a good outcome with no metastatic events being recorded and only one of 98 patients, with a pelvic tumour, succumbing to disease. No patients with tumours in the small bones of the hands and feet died of disease (Fig. 3B). We found that DD CS had a higher frequency of metastatic disease compared with G2/3 disease (G2/3 vs DD CS, *p* = 9e−7). There were no significant differences in the frequency of metastatic or recurrent disease across *IDH1*, *IDH2*, and IDHwt DD CS tumours. However, metastases or recurrent disease appeared to occur less frequently in patients with *IDH2* G2/3 tumours compared to *IDH1* and IDHwt tumours (% metastases/recurrence, *IDH1*: 37%, *IDH2*: 13%, IDHwt: 23%, *IDH1* vs *IDH2*: *p* = 0.04, Fig. 3B). We also found that the time interval between diagnosis and detection of metastatic disease six months following presentation of the primary tumour was shorter in *IDH2* tumours compared to *IDH1* (*p* = 0.04, Fig. 3B). Finally, we found no differences in outcome related to the different *IDH1* mutation contexts (R132C/G/H/L/S) but noted that R132S/L variants found in only a minority number of cases, making statistical analysis difficult (Additional file 3: Supplementary Table 3, Supplementary Fig. 8).

Canonical *TERT* promoter mutations (g.1295113) had an independent hazard ratio (HR) that was equal to that of grade (*TERT*: HR = 2.2, *p* = 0.003, tumour grade: HR = 2.2, *p* = 2e−13, Additional file 3: Supplementary Fig. 8), pointing to the benefit of *TERT* as a biomarker. We also found that overall outcomes were worse in patients whose tumours had *TERT* hypermethylation (*n* = 68, HR = 3.4, *p* = 0.01). Restricting our analyses to high-grade tumours and excluding tumours in the hands and feet, we found that patients whose tumour harboured both *IDH1* and *TERT* mutations had significantly worse outcomes than those with an *IDH1* mutation alone. *TERT* mutations had no effect on outcome in patients with *IDH2* tumours, even though these mutations are found more frequently in combination with *IDH2* mutations (Fig. 3C, Additional file 3: Supplementary Methods). This suggests that *TERT* mutations are context specific and only relevant to outcome predictions in *IDH1* tumours.

Given these findings, identification of tumours with *IDH1* and *TERT* mutations has clinical value.

## Discussion

In this study of patients with CS, involving targeted, whole genome, and methylation data, we provide significant insights into the genetic pathways and dynamics underlying disease progression. Here, with the benefit of large sample numbers, we have been able to study tumours with *IDH1* and *IDH2* mutations independently and shown that they represent distinct genetic and clinical groups.

In addition to confirming that *IDH2* tumours represent the minority group with 14% of cases, we report that they are highly represented in DD CS. They also present as larger tumours and on average over a decade later than *IDH1* tumours. Despite this, *IDH1* and *IDH2* tumours have similar molecular ages suggesting that on average, tumours with *IDH2* mutations have slower cell division rates. Therefore, we speculate that many of these tumours go into growth arrest and become calcified, representing at least a proportion of calcified enchondromas, a lesion commonly detected, when medical imaging is undertaken for unrelated symptoms. This would account for the comparatively lower frequency of *IDH2* tumours. Furthermore, the high incidence of *TERT* mutations and *TERT* promoter methylation in high grade *IDH2* tumours, suggests that these events, through activation of telomerase, have prevented the senescent phenotype and bring about high-grade *IDH2* tumours. This finding could potentially account for the presentation at the relatively late age of these tumours.

No significant differences in the type or number of mutations were identified that accounted for the different clinical findings associated with *IDH1* and *IDH2*. However, we show that *IDH1* tumours are globally more methylated at CpG islands compared to both the *IDH2* and IDHwt tumours. The small numbers of cases studied to date is likely to account for this being unrecognised previously [14, 15]. Indeed, even *IDH1* and *IDH2* gliomas, which are considerably more common than CS, are generally studied together because of their small numbers. Nevertheless, although all *IDH* mutations result in accumulation of 2-HG there is growing evidence that the impact of the different mutations exerts different biological effects. Studies utilising human oligodendroglioma cells have shown that the *IDH1* R132 mutation leads to higher enzymatic activity than that brought about by *IDH2* R172 [56]. Other studies of *IDH1* and *IDH2* mutations in gliomas point to them as having distinct mutational and clinical patterns [57]. Furthermore, different biological effects of 2HG are also seen as a consequence of different *IDH2* mutations [58]. As it is known that 2HG exerts diverse biological functions including regulation of DNA hydroxymethylation, it is feasible that the *IDH1* and *IDH2* mutations explain our different methylation array findings and mediate the different behaviour of the tumour subgroups. However, further research is required to establish this.

The limitation of the study remains the small numbers of cases when broken down by grade, particularly grade 3. Hence, the need to build prospective collaborative studies with detailed clinical outcome over a long period. Previous studies have suggested different effects of *IDH* mutations on clinical outcome in patients with CS [9, 25, 26]. Here, with the benefit of a large patient cohort, we show that although *IDH2* tumours are more commonly associated with *TERT* mutations, only *IDH1* mutations in combination with *TERT* mutations are associated with significantly reduced survival.

## Conclusions

The underlying mutational pathways of tumours with *IDH1* or *IDH2* mutations, or those that are IDHwt, differ significantly. Based on the finding that *TERT* leads to different outcomes in *IDH1-* and *IDH2***-**mutant tumours, we propose that genetic testing for *IDH1*, *IDH2*, and *TERT* promoter mutations in the context of other clinical factors, could be useful in patient stratification.

## Supplementary Information

**Additional file 1:** **Supplementary Tables 1-6**.

**Additional file 2:** **Supplementary Methods**.

**Additional file 3:** **Supplementary Figs. 1-8**, Notes 1-3.

## Data Availability

All ddPCR results are included in Supplementary Table 3. The SNV, indel, and structural calls produced from the WGS data are available in Additional File 1: Supplementary Table 2. The bam and vcf files of the WGS data are covered by the Genomics England participant consent policy, which has a legally obligation to protect personal identity. Raw data available on application for access to Genomics England and the Clinical Interpretation Partnership (GeCIP) and reviewed by the Access Review Committee. Please see https://www.genomicsengland.co.uk/patients-participants/data for further information. The raw methylation array files are available via the ArrayExpress database (https://www.ebi.ac.uk/arrayexpress/experiments/E-MTAB-11031/, accession: E-MTAB-11031). Code is available via the Github page: 10.5281/zenodo.6800685.

## References

[1] Giuffrida AY, Burgueno JE, Koniaris LG, Gutierrez JC, Duncan R, Scully SP (2009). Chondrosarcoma in the United States (1973 to 2003): an analysis of 2890 cases from the SEER database. J Bone Joint Surg Am..

[2] Bovee J, Flanagan AM, Nielsen G, Akihiko Y, Bloem J. The WHO Classification of Tumours Editorial Board. WHO Classification of Tumours: Soft Tissue and Bone Tumours. IARC Press. 2020.

[3] Eefting D, Schrage YM, Geirnaerdt MJA, Le Cessie S, Taminiau AHM, Bovée JVMG (2009). Assessment of interobserver variability and histologic parameters to improve reliability in classification and grading of central cartilaginous tumors. Am J Surg Pathol..

[4] Evans HL, Ayala AG, Romsdahl MM (1977). Prognostic factors in chondrosarcoma of bone: a clinicopathologic analysis with emphasis on histologic grading. Cancer..

[5] Fromm J, Klein A, Baur-Melnyk A, Knösel T, Lindner L, Birkenmaier C (2018). Survival and prognostic factors in conventional central chondrosarcoma. BMC Cancer..

[6] Bovée JV, van der Heul RO, Taminiau AH, Hogendoorn PC (1999). Chondrosarcoma of the phalanx: a locally aggressive lesion with minimal metastatic potential: a report of 35 cases and a review of the literature. Cancer..

[7] Nacev BA, Jones KB, Intlekofer AM, Yu JSE, Allis CD, Tap WD (2020). The epigenomics of sarcoma. Nat Rev Cancer..

[8] Amary MF, Damato S, Halai D, Eskandarpour M, Berisha F, Bonar F (2011). Ollier disease and Maffucci syndrome are caused by somatic mosaic mutations of IDH1 and IDH2. Nat Genet..

[9] Cleven AHG, Suijker J, Agrogiannis G, Briaire-de Bruijn IH, Frizzell N, Hoekstra AS (2017). IDH1 or -2 mutations do not predict outcome and do not cause loss of 5-hydroxymethylcytosine or altered histone modifications in central chondrosarcomas. Clin Sarcoma Res..

[10] Hirata M, Sasaki M, Cairns RA, Inoue S, Puviindran V, Li WY (2015). Mutant IDH is sufficient to initiate enchondromatosis in mice. Proc Natl Acad Sci U S A..

[11] Jin Y, Elalaf H, Watanabe M, Tamaki S, Hineno S, Matsunaga K (2015). Mutant IDH1 dysregulates the differentiation of mesenchymal stem cells in association with gene-specific histone modifications to cartilage- and bone-related genes. PloS One..

[12] Tarpey PS, Behjati S, Cooke S, Van Loo P, Wedge DC, Pillay N (2013). Frequent mutation of the major cartilage collagen gene, COL2A1, in chondrosarcoma. Nat Genet..

[13] Totoki Y, Yoshida A, Hosoda F, Nakamura H, Hama N, Ogura K (2014). Unique mutation portraits and frequent COL2A1 gene alteration in chondrosarcoma. Genome Res..

[14] Guilhamon P, Eskandarpour M, Halai D, Wilson GA, Feber A, Teschendorff AE (2013). Meta-analysis of IDH-mutant cancers identifies EBF1 as an interaction partner for TET2. Nat Commun..

[15] Nicolle R, Ayadi M, Gomez-Brouchet A, Armenoult L, Banneau G, Elarouci N (2019). Integrated molecular characterization of chondrosarcoma reveals critical determinants of disease progression. Nat Commun..

[16] Amary MF, Bacsi K, Maggiani F, Damato S, Halai D, Berisha F (2011). IDH1 and IDH2 mutations are frequent events in central chondrosarcoma and central and periosteal chondromas but not in other mesenchymal tumours. J Pathol..

[17] Amary MF, Ye H, Forbes G, Damato S, Maggiani F, Pollock R (2015). Isocitrate dehydrogenase 1 mutations (IDH1) and p16/CDKN2A copy number change in conventional chondrosarcomas. Virchows Arch Int J Pathol..

[18] Röpke M, Boltze C, Meyer B, Neumann HW, Roessner A, Schneider-Stock R (2006). Rb-loss is associated with high malignancy in chondrosarcoma. Oncol Rep..

[19] Schrage YM, Lam S, Jochemsen AG, Cleton-Jansen A-M, Taminiau AHM, Hogendoorn PCW (2009). Central chondrosarcoma progression is associated with pRb pathway alterations: CDK4 down-regulation and p16 overexpression inhibit cell growth in vitro. J Cell Mol Med..

[20] Bovée JV, van Royen M, Bardoel AF, Rosenberg C, Cornelisse CJ, Cleton-Jansen AM (2000). Near-haploidy and subsequent polyploidization characterize the progression of peripheral chondrosarcoma. Am J Pathol..

[21] Hallor KH, Staaf J, Bovée JVMG, Hogendoorn PCW, Cleton-Jansen A-M, Knuutila S (2009). Genomic profiling of chondrosarcoma: chromosomal patterns in central and peripheral tumors. Clin Cancer Res Off J Am Assoc Cancer Res..

[22] Mandahl N, Johansson B, Mertens F, Mitelman F (2012). Disease-associated patterns of disomic chromosomes in hyperhaploid neoplasms. Genes Chromosomes Cancer..

[23] Olsson L, Paulsson K, Bovée JVMG, Nord KH (2011). Clonal evolution through loss of chromosomes and subsequent polyploidization in chondrosarcoma. PloS One..

[24] Lin Y, Seger N, Chen Y, Hesla AC, Wejde J, Ghaderi M (2018). hTERT promoter mutations in chondrosarcomas associate with progression and disease-related mortality. Mod Pathol Off J U S Can Acad Pathol Inc..

[25] Lugowska I, Teterycz P, Mikula M, Kulecka M, Kluska A, Balabas A (2018). IDH1/2 mutations predict shorter survival in chondrosarcoma. J Cancer..

[26] Zhu GG, Nafa K, Agaram N, Zehir A, Benayed R, Sadowska J (2020). Genomic profiling identifies association of IDH1/IDH2 mutation with longer relapse-free and metastasis-free survival in high-grade chondrosarcoma. Clin Cancer Res Off J Am Assoc Cancer Res..

[27] Prendergast SC, Strobl A-C, Cross W, Pillay N, Strauss SJ, Ye H (2020). Sarcoma and the 100,000 Genomes Project: our experience and changes to practice. J Pathol Clin Res..

[28] Turnbull C, Scott RH, Thomas E, Jones L, Murugaesu N, Pretty FB (2018). The 100 000 Genomes Project: bringing whole genome sequencing to the NHS. BMJ..

[29] Nik-Zainal S, Van Loo P, Wedge DC, Alexandrov LB, Greenman CD, Lau KW (2012). The life history of 21 breast cancers. Cell..

[30] Rausch T, Zichner T, Schlattl A, Stütz AM, Benes V, Korbel JO (2012). DELLY: structural variant discovery by integrated paired-end and split-read analysis. Bioinforma Oxf Engl..

[31] Kumar P, Henikoff S, Ng PC (2009). Predicting the effects of coding non-synonymous variants on protein function using the SIFT algorithm. Nat Protoc..

[32] Ramensky V, Bork P, Sunyaev S (2002). Human non-synonymous SNPs: server and survey. Nucleic Acids Res..

[33] Scrucca L, Fop M, Murphy TB, Raftery AE (2016). mclust 5: clustering, classification and density estimation using Gaussian finite mixture models. R J..

[34] Durinck S, Ho C, Wang NJ, Liao W, Jakkula LR, Collisson EA (2011). Temporal dissection of tumorigenesis in primary cancers. Cancer Discov..

[35] Minussi DC, Nicholson MD, Ye H, Davis A, Wang K, Baker T (2021). Breast tumours maintain a reservoir of subclonal diversity during expansion. Nature..

[36] Alexandrov LB, Kim J, Haradhvala NJ, Huang MN, Tian Ng AW, Wu Y (2020). The repertoire of mutational signatures in human cancer. Nature..

[37] Morris TJ, Butcher LM, Feber A, Teschendorff AE, Chakravarthy AR, Wojdacz TK (2014). ChAMP: 450k chip analysis methylation pipeline. Bioinforma Oxf Engl..

[38] Kolde R. pheatmap: Pretty heatmaps [Software]. URL HttpsCRAN R-Proj Orgpackage Pheatmap. 2015;

[39] Lee DD, Leão R, Komosa M, Gallo M, Zhang CH, Lipman T, et al. DNA hypermethylation within TERT promoter upregulates TERT expression in cancer. J Clin Invest. 129(4):1801. 10.1172/JCI128527 PMC6436876 30932912

[40] Kassambara A, Kosinski M, Biecek P, Fabian S. Survminer: drawing survival curves using ‘ggplot2’. 2021 . Available from: https://CRAN.R-project.org/package=survminer. (Cited 2021 Sep 15)

[41] Pansuriya TC, Oosting J, Verdegaal SHM, Flanagan AM, Sciot R, Kindblom L-G (2011). Maffucci syndrome: a genome-wide analysis using high resolution single nucleotide polymorphism and expression arrays on four cases. Genes Chromosomes Cancer..

[42] Zhang Y, Chen Y, Yang C, Seger N, Hesla AC, Tsagkozis P (2021). TERT promoter mutation is an objective clinical marker for disease progression in chondrosarcoma. Mod Pathol..

[43] Tarpey PS, Behjati S, Young MD, Martincorena I, Alexandrov LB, Farndon SJ, et al. The driver landscape of sporadic chordoma. Nat Commun. 2017;8 Available from: https://www.ncbi.nlm.nih.gov/pmc/articles/PMC5638846/. (cited 2019 Sep 29). 10.1038/s41467-017-01026-0 PMC5638846 29026114

[44] Castresana JS, Barrios C, Gómez L, Kreicbergs A (1992). Amplification of the c-myc proto-oncogene in human chondrosarcoma. Diagn Mol Pathol Am J Surg Pathol Part B..

[45] Morrison C, Radmacher M, Mohammed N, Suster D, Auer H, Jones S (2005). MYC amplification and polysomy 8 in chondrosarcoma: array comparative genomic hybridization, fluorescent in situ hybridization, and association with outcome. J Clin Oncol Off J Am Soc Clin Oncol..

[46] Lindström MS, Jin A, Deisenroth C, White Wolf G, Zhang Y (2007). Cancer-associated mutations in the MDM2 zinc finger domain disrupt ribosomal protein interaction and attenuate MDM2-induced p53 degradation. Mol Cell Biol..

[47] Martincorena I, Raine KM, Gerstung M, Dawson KJ, Haase K, Van Loo P (2017). Universal patterns of selection in cancer and somatic tissues. Cell..

[48] Lucas C-HG, Grenert JP, Horvai A (2021). Targeted next-generation sequencing identifies molecular and genetic events in dedifferentiated chondrosarcoma. Arch Pathol Lab Med..

[49] Lyskjaer I, Lindsay D, Tirabosco R, Steele CD, Lombard P, Strobl A-C (2020). H3K27me3 expression and methylation status in histological variants of malignant peripheral nerve sheath tumours. J Pathol..

[50] Steele CD, Tarabichi M, Oukrif D, Webster AP, Ye H, Fittall M (2019). Undifferentiated sarcomas develop through distinct evolutionary pathways. Cancer Cell..

[51] Campbell PJ, Getz G, Korbel JO, Stuart JM, Jennings JL, Stein LD (2020). Pan-cancer analysis of whole genomes. Nature..

[52] Beird HC, Wu C-C, Ingram DR, Wang W-L, Alimohamed A, Gumbs C (2018). Genomic profiling of dedifferentiated liposarcoma compared to matched well-differentiated liposarcoma reveals higher genomic complexity and a common origin. Mol Case Stud..

[53] O’Malley DP, Opheim KE, Barry TS, Chapman DB, Emond MJ, Conrad EU (2001). Chromosomal changes in a dedifferentiated chondrosarcoma: a case report and review of the literature. Cancer Genet Cytogenet..

[54] Pathmanapan S, Ilkayeva O, Martin JT, Loe AKH, Zhang H, Zhang G-F (2021). Mutant IDH and non-mutant chondrosarcomas display distinct cellular metabolomes. Cancer Metab..

[55] Alexandrov LB, Jones PH, Wedge DC, Sale JE, Campbell PJ, Nik-Zainal S (2015). Clock-like mutational processes in human somatic cells. Nat Genet..

[56] Yan H, Parsons DW, Jin G, McLendon R, Rasheed BA, Yuan W (2009). IDH1 and IDH2 mutations in gliomas. N Engl J Med..

[57] Wang H-Y, Tang K, Liang T-Y, Zhang W-Z, Li J-Y, Wang W (2016). The comparison of clinical and biological characteristics between IDH1 and IDH2 mutations in gliomas. J Exp Clin Cancer Res CR..

[58] Kotredes KP, Razmpour R, Lutton E, Alfonso-Prieto M, Ramirez SH, Gamero AM (2019). Characterization of cancer-associated IDH2 mutations that differ in tumorigenicity, chemosensitivity and 2-hydroxyglutarate production. Oncotarget..

